# Redefining “normal”: discovery of aneuploid epithelial cells in healthy breast tissue

**DOI:** 10.3389/fonc.2025.1558977

**Published:** 2025-07-31

**Authors:** Xiaoyan Chen, Xiaoling Liu, Jiapeng Xue, Zhi Li

**Affiliations:** ^1^ Department of Rehabilitation, Taihe Hospital, Hubei University of Medicine, Shiyan, China; ^2^ Medical College of Nanchang Institute of Technology, Nanchang, Jiangxi, China; ^3^ Department of Pediatric, Taihe Hospital, Hubei University of Medicine, Shiyan, China; ^4^ Department of General Surgery, Taihe Hospital, Hubei University of Medicine, Shiyan, China; ^5^ Interventional Cancer Institute of Chinese Integrative Medicine, Putuo Hospital, Shanghai University of Traditional Chinese Medicine, Shanghai, China

**Keywords:** aneuploid epithelial cells, breast tissue, copy number alterations, cancer, breast cancer

Epithelial cells in breast tissue have long been considered genetically stable, with genetic alterations generally viewed as hallmarks of precancerous lesions or tumor growth. Two new studies published in *Nature* and *Nature Genetics* pose a compelling question: may rare genomically aberrant cells exist within healthy breast tissue? These innovative discoveries not only contest the conventional understanding of breast cancer onset but also pave the way for enhanced early detection and preventive strategies.

A recent study by Lin Y. et al. (1), published in *Nature*, employed single-cell DNA sequencing to examine breast tissue from healthy women and presented the initial evidence of rare aneuploid epithelial cells in normal breast tissue. The research encompassed 83,206 epithelial cells derived from the breast tissue of 49 healthy women, indicating that each woman’s breast tissue harbored infrequent aneuploid epithelial cells (with a median of 3.19%), and this percentage markedly escalated with age. The genomic variation patterns of these aneuploid cells, including increases on chromosome 1q and losses on chromosomes 10q, 16q, and 22q, closely paralleled the genomic hallmarks of invasive breast cancer, suggesting a potential relationship to carcinogenesis. Subsequent spatial mapping research revealed that these cells were primarily situated in the luminal epithelial cells of the breast ducts and lobules, with no presence in the basal myoepithelial cells. Reinforcing these findings, a study by Williams, M.J. et al. published on the same day analyzed 49,238 epithelial cells from 28 women, revealing extensive copy number alterations (2). Crucially, their results showed similarities to the Lin paper, including the ubiquitous nature of CNAs across samples, their near-exclusive presence in luminal epithelial cells, and the frequent involvement of chromosomes 1q and 16q. This independent replication strongly underscores the robustness of these findings. The study highlighted two particularly significant findings. First, in BRCA1/BRCA2 mutation carriers, copy number alterations (CNAs) accumulated to a greater extent than in non-carriers, and these changes were observed even before the loss of heterozygosity (LOH) of the wild-type allele was detectable. Second, across all donor backgrounds, CNA-carrying cells displayed strikingly similar patterns of chromosomal alterations. However, allelic imbalance analyses revealed that these similar outcomes often arose from distinct and independent mutational events.This latter observation provides strong evidence for convergent evolution, suggesting that under selective pressures, a mosaic of aneuploid cells can persist discreetly in normal tissue. These clones may then undergo clonal proliferation under specific conditions, potentially serving as the progenitors from which cancer later develops. The findings indicate that even healthy breast tissue contains a concealed reservoir of genetic aberrations, offering new scientific insights for the early identification and prevention of breast cancer.

These findings reconstitute the genetic stability of “normal” tissues and contest conventional concepts of cancer progression (3–6). Both investigations indicate that genomic aberrant cells are present in healthy breast tissue, which may progressively develop into cancers by clonal expansion and independent mutational occurrences. This study suggests a novel paradigm of tumorigenesis: cancer may arise from latent aneuploid cells within normal tissue that, under specific conditions, undergo carcinogenic transformation and ultimately progress to manifest tumors. This revelation possesses considerable clinical and public health ramifications. It indicates the necessity to reevaluate early cancer screening and prevention methodologies, emphasizing the identification and targeting of genetic aberrant cells in healthy tissues (7, 8). Moreover, the carcinogenic potential of these cells and their interaction with the microenvironment offer new insights for formulating tailored intervention techniques. This research possesses tremendous potential for cancer diagnoses. The advancement of sensitive detection tools for aneuploid cells may enable early tumor identification and customized therapies, hence enhancing patient prognosis dramatically.

While these investigations indicate a possible novel route for breast cancer progression, they possess certain limitations that inform the direction of future research. The limited sample size, predominantly comprising participants from European and American populations, results in insufficient representation of many ethnicities, genetic backgrounds, and environmental factors, thereby restricting the generalizability of current findings. Moreover, most existing data derive from cross-sectional studies, which are incapable of elucidating the temporal evolutionary pathways of aneuploid cells or clarifying the precise mechanism of transition from normal cells to cancer cells. To address this, greater temporal resolution of the evolution of putatively precancerous CNA-altered clones is needed, building upon recent work that has begun to probe these evolutionary histories (9), in order to understand the selective pressures that drive progression. Additionally, although many studies focus on identifying genetic alterations, experimental validation of the *in vivo* functionality of aneuploid cells remains sparse; their specific carcinogenic potential and mechanisms require further exploration. Another notable gap is the paucity of research on interactions between aneuploid cells and the breast microenvironment, particularly concerning how these cells may gain survival advantages and contribute to tumor initiation and progression. To address these limitations, future research should advance in several critical areas. First, large-scale, ethnically diverse longitudinal cohort studies are essential to elucidate the prevalence patterns of aneuploid cells across populations and assess their causal associations with breast cancer. Functional validation is also crucial; *in vitro* experiments and animal models are needed to determine whether these cells demonstrate increased proliferative and invasive capacities, as well as to examine their signaling pathway activity within the tumor microenvironment. Furthermore, the influence of the breast tissue microenvironment—including the role of immune cells in the selective proliferation and carcinogenic potential of aneuploid cells—warrants understanding investigation. The application of novel technologies, such as single-cell sequencing, liquid biopsy, and the examination of milk and other breast secretions (10, 11), offers promise for translating fundamental research into clinical practice, enabling accurate early detection and timely intervention for high-risk individuals (12, 13). Finally, continuing to expand similar research frameworks to the normal tissues of other organs may reveal how common of a phenomenon genetically aberrant cells are and elucidate their roles in the genesis of other cancer types (14, 15).

In summary, these two investigations have discovered rare aneuploid epithelial cells in normal breast tissue, contesting conventional theories of cancer formation and reshaping our comprehension of genetic stability in “normal” tissues. The copy number variations present in these cells resemble those found in invasive breast cancer, indicating they may signify initial stages of carcinogenesis, despite the lack of histological abnormalities. This line of study bears substantial implications not only for understanding the beginnings of breast cancer but also for creating new routes for early detection, risk assessment, and preventive therapies.

## References

[1] Lin Y Wang J Wang K Bai S Thennavan A Wei R. Normal breast tissues harbour rare populations of aneuploid epithelial cells. Nature. (2024) 56(12):2753–62. doi: 10.1038/s41586-024-08129-x, PMID: 39567687 PMC12367288

[2] Williams MJ Oliphant MUJ Au V Liu C Baril C O'Flanagan C. Luminal breast epithelial cells of BRCA1 or BRCA2 mutation carriers and noncarriers harbor common breast cancer copy number alterations. Nat Genet. (2024) 56(12):2753–62. doi: 10.1038/s41588-024-01988-0, PMID: 39567747 PMC11631757

[3] Curtis C Shah SP Chin SF Turashvili G Rueda OM Dunning MJ. The genomic and transcriptomic architecture of 2,000 breast tumours reveals novel subgroups. Nature. (2012) 486:346–52. doi: 10.1038/nature10983, PMID: 22522925 PMC3440846

[4] Cereser B Yiu A Tabassum N Del Bel Belluz L Zagorac S Ancheta KRZ. The mutational landscape of the adult healthy parous and nulliparous human breast. Nat Commun. (2023) 14:5136. doi: 10.1038/s41467-023-40608-z, PMID: 37673861 PMC10482899

[5] Li Y Roberts ND Wala JA Shapira O Schumacher SE Kumar K. Patterns of somatic structural variation in human cancer genomes. Nature. (2020) 578:112–21. doi: 10.1038/s41586-019-1913-9, PMID: 32025012 PMC7025897

[6] Lips EH Kumar T Megalios A Visser LL Sheinman M Fortunato A. Genomic analysis defines clonal relationships of ductal carcinoma in *situ* and recurrent invasive breast cancer. Nat Genet. (2022) 54:850–60. doi: 10.1038/s41588-022-01082-3, PMID: 35681052 PMC9197769

[7] Gao T Kastriti ME Ljungström V Heinzel A Tischler AS Oberbauer R. A pan-tissue survey of mosaic chromosomal alterations in 948 individuals. Nat Genet. (2023) 55:1901–11. doi: 10.1038/s41588-023-01537-1, PMID: 37904053 PMC10838621

[8] Cortés-Ciriano I Lee JJ Xi R Jain D Jung YL Yang L. Comprehensive analysis of chromothripsis in 2,658 human cancers using whole-genome sequencing. Nat Genet. (2020) 52:331–41. doi: 10.1038/s41588-019-0576-7, PMID: 32025003 PMC7058534

[9] Nishimura T Kakiuchi N Yoshida K Sakurai T Kataoka TR Kondoh E. Evolutionary histories of breast cancer and related clones. Nature. (2023) 620:607–14. doi: 10.1038/s41586-023-06333-9, PMID: 37495687 PMC10432280

[10] Holmquist DG Papanicolaou GN. The exfoliative cytology of the mammary gland during pregnancy and lactation. Ann N Y Acad Sci. (1956) 63:1422–35. doi: 10.1111/j.1749-6632.1956.tb32147.x, PMID: 13314484

[11] Bhat-Nakshatri P Kumar B Simpson E Ludwig KK Cox ML Gao H. Breast cancer cell detection and characterization from breast milk-derived cells. Cancer Res. (2020) 80:4828–39. doi: 10.1158/0008-5472.CAN-20-1030, PMID: 32934021 PMC7642166

[12] Watson EV Lee JJ Gulhan DC Melloni GEM Venev SV Magesh RY. Chromosome evolution screens recapitulate tissue-specific tumor aneuploidy patterns. Nat Genet. (2024) 56:900–12. doi: 10.1038/s41588-024-01665-2, PMID: 38388848 PMC11096114

[13] Knouse KA Lopez KE Bachofner M Amon A. Chromosome segregation fidelity in epithelia requires tissue architecture. Cell. (2018) 175:200–211.e13. doi: 10.1016/j.cell.2018.07.042, PMID: 30146160 PMC6151153

[14] Lee-Six H Olafsson S Ellis P Osborne RJ Sanders MA Moore L. The landscape of somatic mutation in normal colorectal epithelial cells. Nature. (2019) 574:532–7. doi: 10.1038/s41586-019-1672-7, PMID: 31645730

[15] Martincorena I Roshan A Gerstung M Ellis P Van Loo P McLaren S. Tumor evolution. High burden and pervasive positive selection of somatic mutations in normal human skin. Science. (2015) 348:880–6. doi: 10.1126/science.aaa6806, PMID: 25999502 PMC4471149

